# Arsenic trioxide disrupts glioma stem cells via promoting PML degradation to inhibit tumor growth

**DOI:** 10.18632/oncotarget.5836

**Published:** 2015-10-14

**Authors:** Wenchao Zhou, Lin Cheng, Yu Shi, Susan Q. Ke, Zhi Huang, Xiaoguang Fang, Cheng-wei Chu, Qi Xie, Xiu-wu Bian, Jeremy N. Rich, Shideng Bao

**Affiliations:** ^1^ Department of Stem Cell Biology and Regenerative Medicine, Lerner Research Institute, Cleveland Clinic, Cleveland, OH 44195, USA; ^2^ State Key Laboratory of Medical Genomics, Shanghai Institute of Hematology, Rui Jin Hospital, Shanghai Jiao Tong University, Shanghai 200025, China; ^3^ Institute of Pathology and Southwest Cancer Center, Southwest Hospital, Third Military Medical University, Chongqing 400038, China

**Keywords:** glioblastoma, glioma stem cell, arsenic trioxide, PML, c-Myc

## Abstract

Glioblastoma multiforme (GBM) is the most lethal brain tumor. Tumor relapse in GBM is inevitable despite maximal therapeutic interventions. Glioma stem cells (GSCs) have been found to be critical players in therapeutic resistance and tumor recurrence. Therapeutic drugs targeting GSCs may significantly improve GBM treatment. In this study, we demonstrated that arsenic trioxide (As_2_O_3_) effectively disrupted GSCs and inhibited tumor growth in the GSC-derived orthotopic xenografts by targeting the promyelocytic leukaemia (PML). As_2_O_3_ treatment induced rapid degradation of PML protein along with severe apoptosis in GSCs. Disruption of the endogenous PML recapitulated the inhibitory effects of As_2_O_3_ treatment on GSCs both *in vitro* and in orthotopic tumors. Importantly, As_2_O_3_ treatment dramatically reduced GSC population in the intracranial GBM xenografts and increased the survival of mice bearing the tumors. In addition, As_2_O_3_ treatment preferentially inhibited cell growth of GSCs but not matched non-stem tumor cells (NSTCs). Furthermore, As_2_O_3_ treatment or PML disruption potently diminished c-Myc protein levels through increased poly-ubiquitination and proteasome degradation of c-Myc. Our study indicated a potential implication of As_2_O_3_ in GBM treatment and highlighted the important role of PML/c-Myc axis in the maintenance of GSCs.

## INTRODUCTION

Glioblastoma multiforme (GBM), classified as the grade IV astrocytoma, is the most lethal and common type of primary brain tumor. With a crude annual incidence of 3 per 100,000 individuals, GBM predicts a median survival of less than 16 months associated with very poor life quality [1]. Despite aggressive therapies including surgical resection, radiation and chemotherapy, tumor relapse is a common event in GBM patients [1]. Recent studies indicate that a subset of GBM tumor cells with stem cell-like properties, named glioma stem cells (GSCs), play a critical role in therapeutic resistance, tumor recurrence and malignant progression [2–7]. At the apex of the differentiation hierarchy of glioma cells, GSCs have the capacities to self-renew, differentiate, and recapitulate the whole tumor [8]. GSCs showed potent tumor formation ability in immunocompromised mice [2, 3]. GSCs are also more resistant to irradiation relative to matched non-stem tumor cells (NSTCs) [3, 9, 10]. An increase of GSC population was observed *in vivo* after chemo-radiation treatment, further supporting the involvement of GSCs in therapeutic resistance and the resultant tumor relapse [3, 11–13]. In addition, GSCs promote tumor angiogenesis, pericyte derivation, cancer invasion, and immune evasion, all contributing to the treatment failure [6, 14–17]. Therefore, efficient elimination of the GSC population is a critical step to achieve successful GBM treatment.

Multiple drugs have been applied in GBM treatment, but most of them generate only mild and temporary beneficial outcomes. Addition of Temozolomide (TMZ) to ionic irradiation (IR) statistically improves the prognosis of newly diagnosed GBM patients, but the overall survival rate after treatment is still very poor [18]. The limited effect of TMZ treatment can largely be ascribed to the GSC population. Genetic depletion of the Nestin-positive GSCs restored the response of GBM tumors to TMZ in the genetically engineered mouse model [7]. In fact, exposure to TMZ resulted in expansion of GSC population either by selective amplification of GSCs or by phenotypic shift of non-stem tumor cells to a GSC-like state [19]. In addition, the anti-VEGF-A monoclonal antibody bevacizumab targeting tumor vascularization has a transient inhibition on GBM tumor growth, but the effect is greatly attenuated in the GSC population due to the VEGFR2-Neuropilin-1 autocrine loop [20]. Furthermore, inhibition of vessel formation will cause hypoxia which in the long run will facilitate GSC maintenance or expansion [21–23]. Although numerous efforts have been put in exploration of new drugs targeting GSCs to control GBM tumors, so far no obvious advance has been made.

Arsenic trioxide (As_2_O_3_) is a small molecular drug approved by FDA for leukemia treatment [24]. During the development of acute promyelocytic leukemia (APL), the PML-RARα fusion protein has been demonstrated to underlie the abnormal transcription and the consequent rapid growth of tumor cells [25]. Administration of As_2_O_3_ in leukemia induces the ubiquitination-mediated degradation of the PML-RARα fusion protein via multiple pathways and manifests substantial therapeutic effects [26–29]. Furthermore, elimination of PML-RARα by As_2_O_3_ treatment clears leukemia-initiating cells in mouse APL, suggesting the potential of As_2_O_3_ in targeting cancer stem cells [30]. So far, no PML-RARα mutant has been reported in GBM. However, recent studies demonstrated that the As_2_O_3_ target PML itself plays a critical role in the maintenance of leukemia initiating cells in chronic myeloid leukemia [31]. This discovery indicates the potential application of As_2_O_3_ in treating other cancers such as GBM bearing cancer stem cells. In fact, preliminary studies suggested the inhibitory effect of As_2_O_3_ on *in vitro* cultured glioma tumour-spheres [32], but the consequences of As_2_O_3_ administration on GSC-derived GBMs *in vivo* as well as the underlying molecular mechanisms remained largely unknown.

Inspired by the new breakthrough in targeting cancer stem cells by As_2_O_3_ in several types of leukemia [30, 31], we examined the effect of As_2_O_3_ on GSCs *in vitro* and in GSC-derived xenografts. As_2_O_3_ treatment showed a dramatic inhibition on GSC growth in culture and tumor progression in GBM xenografts. Moreover, As_2_O_3_ treatment diminished PML protein in GSCs. Consistently, knockdown of PML had similar outcomes as As_2_O_3_ treatment, suggesting that As_2_O_3_ targets GSCs via degradation of PML protein. In contrast, As_2_O_3_ treatment displayed negligible effect on non-stem glioma cells. Finally, we found that c-Myc is one of the key downstream effectors in response to the As_2_O_3_-mediated PML degradation in GSCs. Our findings indicate that ablation of cancer stem cells in GBM by As_2_O_3_ treatment may have therapeutic potential and clinical implication in the control of this lethal cancer.

## RESULTS

### As_2_O_3_ treatment inhibited GSC sphere formation and tumor growth

**Figure 1:**
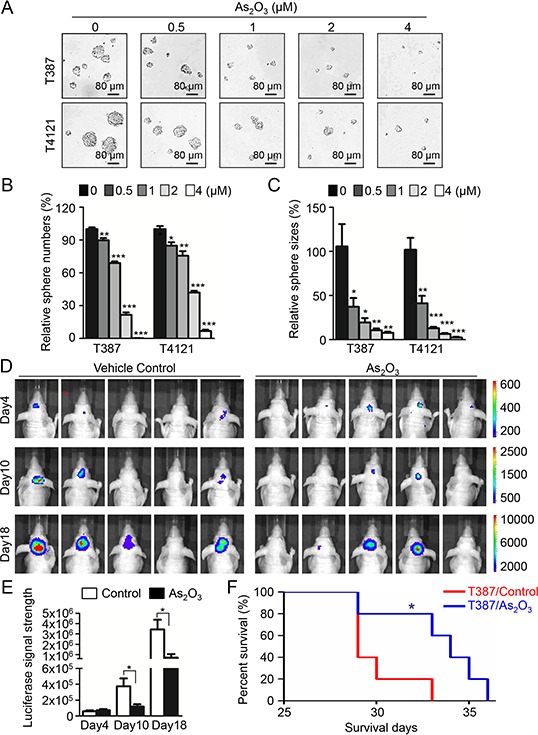
As_2_O_3_ treatment effectively inhibited GSC tumorsphere formation and tumor growth and significantly increased survival of mice bearing GSC-derived xenografts **A.** Representative images of GSC tumorspheres showing that As_2_O_3_ treatment suppressed GSC tumorsphere formation in a dose-dependent manner. T387 or T4121 GSCs were planted in 96 well plates at the density of 2,000 cells per well for 24 hours, and then treated with varied concentration of As_2_O_3_ as indicated for 48 hours. **B, C.** Statistical bar graphs showing the inhibitory effects of different doses of As_2_O_3_ treatment on GSC tumorsphere numbers B. and sizes C. Data represent three independent experiments. *p < 0.05; **p < 0.001; ***p < 0.001 (mean ± s.e.m.; two tailed unpaired *t*-test). **D.** *In vivo* bioluminescent imaging of GSC-derived intracranial tumor growth in the As_2_O_3_ treated and control mice. T387 GSCs (500 cells) transduced with luciferase were transplanted into brains of immunocompromised mice to establish orthotopic GBM xenografts. Mice were treated with As_2_O_3_ (5 μg/g) or vehicle control daily through IP injection starting day 4 after GSC transplantation. Representative images of luciferase signal in mouse brains on Days 4, 10 and 18 after GSC implantation were shown. **E.** Bar graphs of the average signal of luciferase activity in As_2_O_3_ treated and control mouse brains in D showing that As_2_O_3_ treatment significantly inhibited GSC tumor growth. *p < 0.05 (mean ± s.e.m.; two tailed unpaired *t*-test). **F.** Kaplan–Meier survival curves of mice bearing GSC-derived xenografts after As_2_O_3_ or vehicle treatment. Logrank analysis revealed the significant extension of mouse survival in As_2_O_3_-treated group relative to the control group. (*n* = 5 for each group; *: *p* < 0.05).

To examine the putative effect of As_2_O_3_ treatment on GSCs *in vitro*, we performed the tumorsphere formation assays with GSCs isolated from GBM surgical specimens or xenografts. Sorted GSCs (T4121 or T387) were cultured in 6-well plates and treated with various doses of As_2_O_3_. Treatment with As_2_O_3_ (1–4 μM) effectively inhibited GSC sphere formation and caused a significant decrease both in sphere size and number (Figure 1A–1C). In detail, treatment of GSCs with As_2_O_3_ at 2 μM resulted in loss of 50–70% spheres and the treatment with 4 μM As_2_O_3_ markedly inhibited GSC sphere formation (Figure 1A–1C). Even at the dosage of 0.5 μM, the effect of As_2_O_3_ treatment was very significant with a 50% decrease on sphere size (Figure 1C). Consistently, cell titer assay confirmed that As_2_O_3_ treatment (1–4 μM) significantly suppressed GSC growth *in vitro* (data not shown). Thus, As_2_O_3_ treatment has potent inhibitory effect on GSC tumorsphere formation and growth *in vitro*.

We then examined the impact of As_2_O_3_ administration on tumor growth of the GSC-derived orthotopic xenografts. Mice bearing GBM xenografts derived from luciferase-expressing GSCs (T387) were treated with As_2_O_3_ (5 μg/g) [33, 34] or vehicle control daily from the fourth day after GSC implantation. *In vivo* tumor growth after treatment was determined by the luciferase activity in tumors at indicated time points. While the initiating tumor sizes were similar at Day 4 after GSC implantation, the delayed tumor growth was detected in the As_2_O_3_-treated group, which was represented by the significant less luciferase signal in mouse brains in As_2_O_3_-treated mice than control mice at Day 10 and Day 18 (Figure 1D and 1E). As a result of the inhibited tumor growth, mice treated with As_2_O_3_ also displayed a significantly extended survival relative to the control mice (Figure 1F). These data demonstrate that As_2_O_3_ treatment significantly inhibited tumor growth of GSC-derived xenografts and increased survival of animals bearing the GBM xenografts.

### As_2_O_3_ treatment reduced PML protein and promoted apoptosis in GSCs

**Figure 2:**
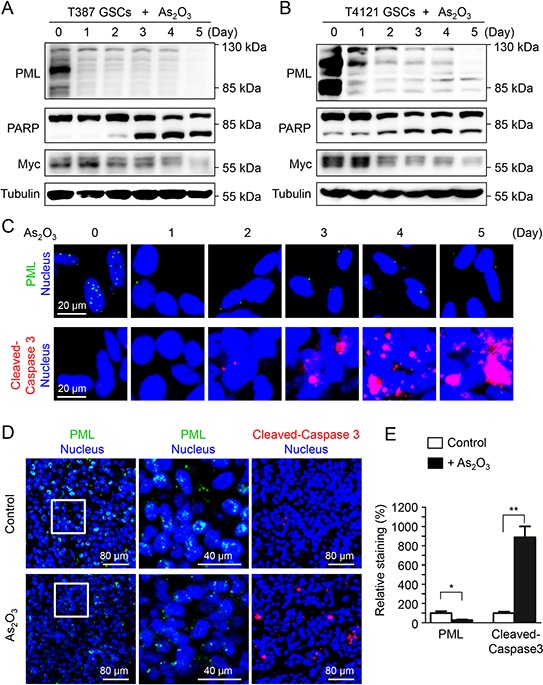
As_2_O_3_ treatment reduced PML protein and promoted apoptosis in GSCs **A, B.** Immunoblot analysis of GSCs (T387 and T4121) treated with As_2_O_3_ for varied duration (0–5 days) as indicated. A decrease of PML protein levels along with an increase of the apoptotic marker cleaved PARP was detected after As_2_O_3_ treatment. **C.** Immunofluorescent staining of PML and cleaved Caspase-3 (an apoptotic marker) in GSCs (T387) treated with As_2_O_3_ for varied duration. A decreased PML signal (green) along with an increased cleaved Caspase-3 (red) was detected after As_2_O_3_ treatment. **D.** Immunofluorescent staining of PML (green) and cleaved Caspase-3 (red) on frozen sections of GSC-derived intracranial xenografts (T387) treated with As_2_O_3_ or vehicle control. The sections were counterstained with DAPI for nuclei (blue). As_2_O_3_ treatment resulted in decreased PML level (green) and increased cleaved-Caspase-3 signal (red). **E.** Statistical bar graphs showing a significant decrease of PML signal and the significant increase of cleaved-Caspase-3 in As_2_O_3_-treated xenografts relative to the control tumors. PML signals were quantified according to the size of the area stained by the anti-PML antibody. Cleaved-Caspase-3 signals were quantified according to the numbers of dots stained by the corresponding antibody. Image J was applied for the quantification. *p < 0.05; **p < 0.01 (mean ± s.e.m.; two tailed unpaired *t*-test).

Previous reports demonstrated that As_2_O_3_ treatment triggered an immediate downregulation of PML [26, 27], therefore we explored the dynamic of PML protein in GSCs in response to As_2_O_3_ treatment. A time course study showed that As_2_O_3_ treatment induced a gradual loss of PML protein in T387 and T4121 GSCs (Figure 2A and 2B, top panels). Meanwhile, the cleaved PARP, an apoptotic marker, increased along with the PML reduction during the course of As_2_O_3_ treatment (Figure 2A and 2B, middle panels), suggesting the induction of GSC apoptosis by As_2_O_3_ treatment. This result was further confirmed by immunofluorescent staining of another apoptotic marker, the cleaved Caspase-3. As shown in Figure 2C, As_2_O_3_ treatment resulted in an increase of the cleaved Caspase-3 along with a gradual decrease of PML protein levels. These data indicate that induction of GSC apoptosis may underlie the inhibited cell growth after As_2_O_3_ treatment. As As_2_O_3_ treatment *in vivo* inhibited GSC tumor growth, we further examined the effect of As_2_O_3_ on PML protein and apoptosis in GSC-derived xenografts. Immunofluorescent staining demonstrated a significant decrease of PML signals accompanied by an increase of the cleaved Caspase-3 staining in the As_2_O_3_-treated xenografts relative to the control xenografts (Figure 2D and 2E). Collectively, these data demonstrate that As_2_O_3_ treatment reduced PML protein and induces apoptotic cell death *in vitro* and in GSC-derived xenografts.

### PML knockdown in GSCs mimics As_2_O_3_ treatment *in vitro* and *in vivo*

**Figure 3:**
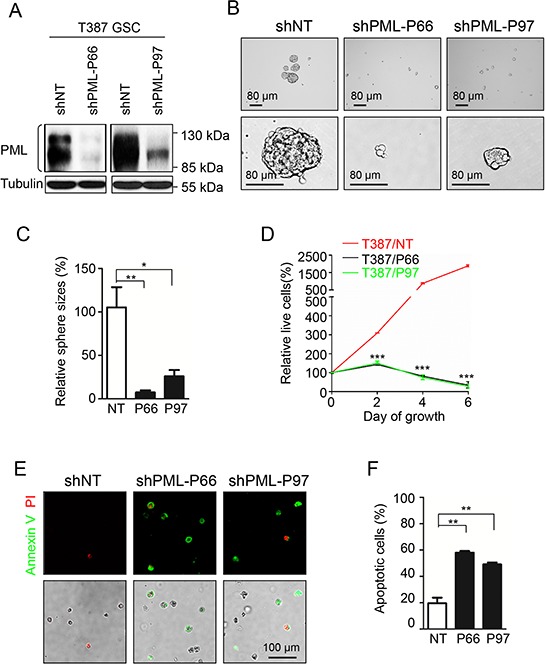
Knock-down of PML in GSCs mimics As_2_O_3_ treatment to inhibit GSC growth *in vitro* **A.** Immunoblot analysis showing the efficiency of PML knockdown in GSCs by shPML lentiviruses. Both shPML-P66 and shPML-P97 infection reduced more than 80 percent of endogenous PML in GSCs. **B.** Knock-down of PML reduced GSC sphere formation. GSCs (T387) were infected with shPML or shNT lentiviruses for 24 hours, and then planted into 96 well plates at the concentration of 2,000 cells per well. Representative images showing GSC spheres 96 hours after lentiviral infection. A dramatic reduction of GSC sphere size was observed after PML knockdown. **C.** Statistical bar graphs showing a significant decrease in tumorsphere sizes of shPML-expressing GSCs relative to shNT-expressing GSCs. *p < 0.05; **p < 0.01 (mean ± s.e.m.; two tailed unpaired *t*-test). **D.** Cell titer assays showing cell growth of GSCs transduced shPML or shNT control. GSCs were infected with shPML or shNT lentiviruses for 24 hours, and then split into 96 well plates at the concentration of 2,000 cells per well. Cell titer was determined by the Glo luminescent cell viability assay kit (Promega) at the indicated time points. Disruption of PML significantly inhibited GSC growth and induced cell death. ***p < 0.001 (mean ± s.e.m.; two tailed unpaired *t*-test). **E.** Annexin V (an apoptosis marker) staining of GSCs transduced with shPML or shNT. 48 hours after infection with shPML or shNT lentiviruses, cells were trypsinized and stained with Annexin V apoptosis detection kit (BD Pharmingen). GSCs transduced with shPML showed more apoptotic cells with Annexin V staining than the control GSCs with shNT. **F.** Statistical bar graphs showing a significant increase of apoptosis in shPML-expressing GSCs relative to the shNT-expressing GSCs measured by Annexin V staining. **p < 0.01 (mean ± s.e.m.; two tailed unpaired *t*-test).

**Figure 4:**
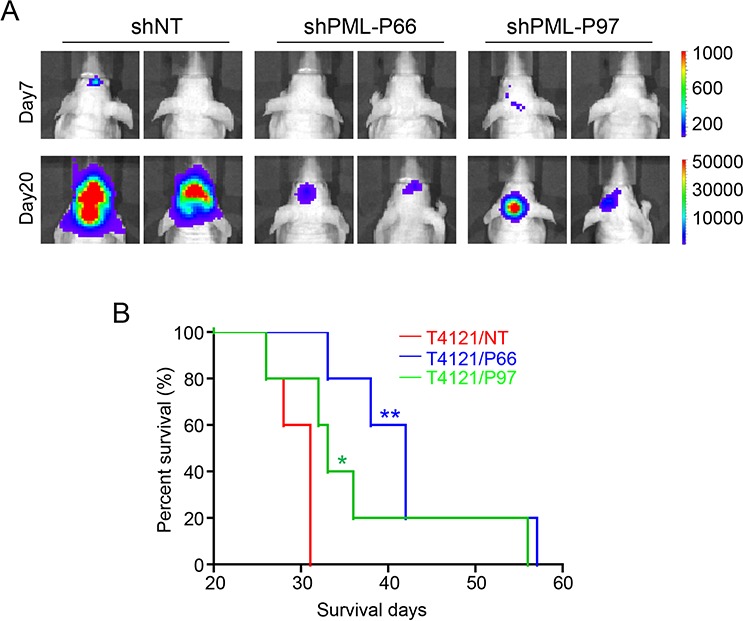
Disruption of PML in GSCs inhibits GSC-derived GBM tumor growth *in vivo* **A.** *In vivo* bioluminescent imaging to monitor the tumor growth of orthotopic xenografts derived from shPML- and shNT-expressing GSCs. GSCs (T387) transduced with shPML or shNT and luciferase were transplanted into brains of immunocompromised mice (5,000 cells/mouse) through intracranial injection. Luciferase signals were monitored at indicated time points after GSC transplantation. Representative luciferase images on days 7 and 20 showed that much weaker luciferase signals were detected in xenografts derived from shPML-expressing GSCs than that derived from shNT-expressing GSCs, indicating a retarded tumor growth by disruption of PML. **B.** Kaplan–Meier survival curves of mice bearing GBM xenografts derived from shPML- or shNT- expressing GSCs. Mice intracranially implanted with shPML- and shNT-expressing GSCs were monitored and maintained until the manifestation of neurological signs. Logrank analysis revealed a significant extension of survival in groups of mice bearing shPML-GSC-derived xenografts relative to the control mice bearing shNT-GSC-derived xenografts. (*n* = 5 mice for each group; *p < 0.05; **p < 0.01).

As As_2_O_3_ treatment led to PML reduction and induced apoptosis in GSCs, we next sought to determine whether As_2_O_3_ exerts its effects on GSCs through downregulation of PML. To address this issue, we examined if disruption of PML by shRNA in GSCs shows similar effects as As_2_O_3_ treatment. Two non-overlapping shRNAs targeting PML, named as shPML-P66 and shPML-P97, were applied in this study to mediate PML knockdown. Immunoblot validated the efficiency of the shRNAs in GSCs (T387 and D456) with more than 80% decrease of endogenous PML protein (Figure 3A, Supplementary Figure S1A). Tumorsphere formation assays of the GSCs infected with non-targeting (shNT) or shPML lentiviruses showed a significant inhibition of GSC sphere formation after PML knockdown (Figure 3B and 3C, Supplementary Figure S1B). The inhibitory effect of PML disruption on GSC growth was further quantified by cell titer assays. Interestingly, PML knockdown not only suppressed GSC proliferation but also caused severe cell death, which was demonstrated by the downward cell titer curves in cells infected with shPML viruses (Figure 3D, Supplementary Figure S1C). Staining of the apoptotic marker Annexin V confirmed the occurrence of massive apoptosis in GSCs two days after infection of shPML viruses (Figure 3E and 3F), suggesting that increased GSC cell death may be a result of apoptosis induced by PML disruption. Furthermore, disruption of PML by the shRNAs severely impaired tumor growth of the GSC-derived xenografts (Figure 4A). As a consequence, mice bearing the GSC-derived xenografts expressing shPML had a significantly extended survival relative to the control group (Figure 4B, Supplementary Figure S1D). Taken together, PML disruption resulted in increased apoptosis and reduced tumorigenic capacity of GSCs, which recapitulates the effects of As_2_O_3_ treatment *in vitro* and *in vivo*.

### As_2_O_3_ treatment reduced GSC population in GBM xenografts

**Figure 5:**
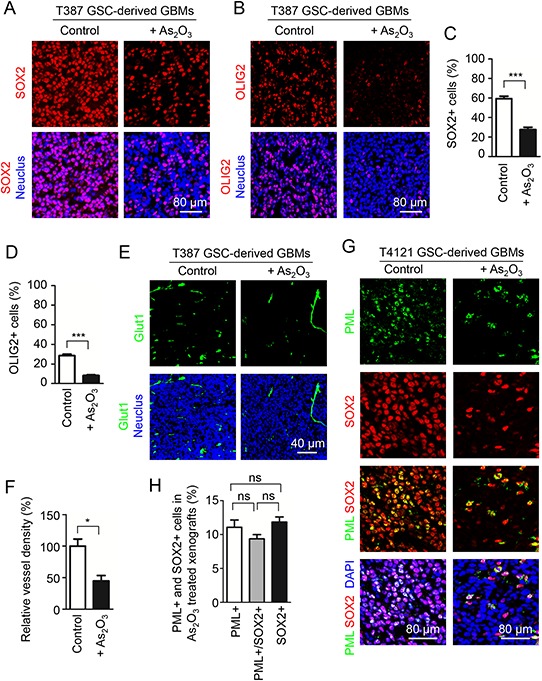
As_2_O_3_ treatment reduced PML protein and GSC population *in vivo* **A, B.** Immunofluorescent staining of the GSC markers SOX2 (A, in red) or OLIG2 (B, in red) on frozen sections of GSC-derived intracranial xenografts from mice treated with As_2_O_3_ or the vehicle control. 4 days after GSC transplantation, mice bearing the GSC-derived xenografts (T387) were treated with As_2_O_3_ (5 μg/g) or vehicle control daily through IP injection for 15 days, and then mouse brains bearing the tumors were harvested and sectioned for the immunofluorescence. As_2_O_3_ treatment markedly reduced SOX2+ or OLIG2+ (GSCs) in the GSC-derived xenografts. **C, D.** Statistical bar graphs showing a significant decrease in SOX2+ C. or OLIG2+ D. population in the As_2_O_3_-treated xenografts relative to the control xenografts. ***p < 0.001 (mean ± s.e.m.; two tailed unpaired *t*-test). **E.** Immunofluorescent staining of the endothelial marker Glut1 detecting blood vessels (in green) on frozen sections of xenografts from mice treated with As_2_O_3_ or vehicle control. Sections were counterstained with DAPI to label nuclei (blue). Xenografts from mice treated with As_2_O_3_ showed lower vessel density relative to the control xenografts. **F.** Statistical bar graphs showing a significant decrease in vessel density (Glut1+) in the As_2_O_3_-treated xenografts relative to the control xenografts. *p < 0.05 (mean ± s.e.m.; two tailed unpaired *t*-test). **G.** Immunofluorescent staining of PML (in green) and the GSC marker SOX2 (in red) in T4121 GSC-derived xenografts from mice treated with As_2_O_3_ or vehicle control. Frozen tumor sections were counterstained with DAPI (blue). As_2_O_3_-treated xenografts displayed much fewer PML+ cells and SOX2+ cells (GSCs). Noticeably, most PML staining signal overlapped with SOX2 signal in GSCs in As_2_O_3_-treated xenografts. **H.** Bar graphs showing percentages of PML positive cells and the SOX2 positive GSC population in xenografts treated with As_2_O_3_. The majority of SOX2 positive GSCs remaining in the As_2_O_3_-treated xenografts were also PML positive.

As As_2_O_3_ treatment mimics PML disruption to induce GSC apoptosis *in vitro*, we further investigated the effect of As_2_O_3_ treatment on GSC population in GSC-derived xenografts. Immunofluorescent staining of GSC markers SOX2 and OLIG2 demonstrated that As_2_O_3_ treatment dramatically reduced GSC population in the T387 and T4121 GSC-derived xenografts (Figure 5A–5D, Supplementary Figure S2A–S2D). GSCs express high levels of the angiogenic factor VEGF to promote tumor angiogenesis [16, 35], and As_2_O_3_-induced GSC disruption in the orthotopic tumors is supposed to influence the tumor vascularization. We next examined whether As_2_O_3_-induced reduction of GSCs impacts vessel density in GBM xenografts. Immunofluorescent staining of the vessel endothelial cell marker Glut1 indicated that As_2_O_3_ treatment significantly reduced vessel density in GSC-derived xenografts (Figure 5E and 5F). Thus, As_2_O_3_ treatment reduced GSC population in GBMs, which may in turn attenuate angiogenic factors produced by GSCs to inhibit tumor vascularization. Consistently, As_2_O_3_ treatment reduced PML protein and SOX2-positive cells (GSCs) in xenografts derived from two GSC lines (T387 and T4121) (Figure 5G, Supplementary Figure S2E). Although some cancer cells in the As_2_O_3_-treated xenografts remained to be SOX2 positive (GSCs), these SOX2 signal almost overlapped with the PML staining (Figure 5G, Supplementary Figure S2E), and the majority of SOX+ cells (GSCs) were also PML-positive (Figure 5H, Supplementary Figure S2F). Collectively, these data suggested that degradation of PML triggered by As_2_O_3_ resulted in the reduction of GSC population after As_2_O_3_ treatment *in vivo*, supporting a critical role of PML in the maintenance of GSCs in GBM tumors.

### GSCs and matched NSTCs displayed distinct PML distributions and showed different responses to As_2_O_3_ treatment

Since disruption of PML effectively induced apoptosis of GSCs, we sought to determine whether the effect of As_2_O_3_ on GSCs is preferential. PML has been shown to be the organizer for certain specific nuclear structures, i.e. nuclear bodies, which are punctate small dots within nuclei. Nuclear bodies are thought to be transcriptional hot spots and may be involved in many critical cellular processes [36]. Thus, PML distribution and nuclear pattern are closely associated with its functional status [37–39]. To further address the functional significance of PML in GSCs, we examined PML distribution in GSCs and matched NSTCs by immunofluorescent staining. Grossly, PML signals were exclusively detected in nuclei in both GSCs and NSTCs. However, while PML nuclear dots appeared to be smaller and sharper in GSCs, the PML dots were larger and vaguer in NSTCs (Supplementary Figure S3). The smeared PML distribution in NSTCs may represent a less organized status.

**Figure 6:**
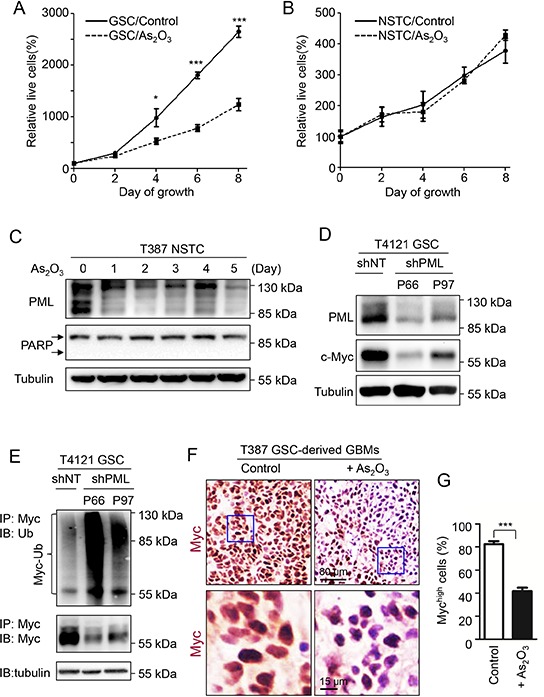
c-Myc is the downstream effector of PML in GSCs that associates with the GSC-preferential effects of As_2_O_3_ **A, B.** Cell growth curves of T387 GSCs (A) and matched NSTCs (B) in response to As_2_O_3_ treatment. 2,000 GSCs or NSTCs were planted in each well of 96 well plates and then treated with 1 μM As_2_O_3_ or vehicle control (0.01N NaOH). Cell titer was determined by the Glo luminescent cell viability assay kit (Promega) at the indicated time points. As_2_O_3_ treatment significantly inhibited cell growth of GSCs but not matched NSTCs. *p < 0.05; ***p < 0.001 (mean ± s.e.m.; two tailed unpaired *t*-test). **C.** Immunoblot analysis of NSTCs treated with As_2_O_3_ for varied duration. As_2_O_3_ treatment resulted in reduced PML protein but did not induce the cleaved PARP, a marker of apoptosis. **D.** Immunoblot analysis of PML and c-Myc after PML knockdown by shPML (P66 or P97) in GSCs. Disruption of endogenous PML by shPML resulted in a substantial decrease in c-Myc protein levels in GSCs (T4121). **E.** Ubiquitination assay of c-Myc in GSCs transduced with shPML (P66 or P97) or shNT control. GSCs (T4121) were transfected with shPML or shNT through lentiviral infection for 36 hours, and then treated with 20 μM MG132 for 6 hours. Cell lysate was subjected to immunoprecipitation with the anti-c-Myc agarose beads, and the c-Myc poly-ubiquitination status was determined with anti-ubiquitin antibody. A significant increase in c-Myc polyubiquitination and a decrease of c-Myc protein levels were detected in cells transduced with shPML relative to the shNT control. Tubulin was used as the internal control for the input of immunoprecipitation (lower panel). **F.** Immunohistochemistry of c-Myc to determine the population of cells with high c-Myc expression in T387 GSC-derived xenografts after As_2_O_3_ treatment. Sections of GSC-derived xenografts were stained with the antibody against c-Myc. Xenografts treated with As_2_O_3_ showed much weaker c-Myc staining (in brown) than those treated with the vehicle control. **G.** Bar graphs showing the population of cells with high c-Myc expression in the xenografts treated with As_2_O_3_ or vehicle control. A significant reduction of cells with high c-Myc expression was detected in As_2_O_3_-treated xenografts relative to the control xenografts. ***p < 0.001 (mean ± s.e.m.; two tailed unpaired *t*-test).

As GSCs and NSTCs displayed different patterns of PML nuclear bodies, we examined whether GSCs and NSTCs show differential sensitivity to As_2_O_3_ treatment. While As_2_O_3_ treatment significantly inhibited GSC cell growth, the same dose of As_2_O_3_ treatment did not show a significant effect on matched NSTCs (Figure 6A and 6B, Supplementary Figure S4A and S4B). Consistently, no induction of the cleaved PARP (the apoptotic marker) was observed in NSTCs after As_2_O_3_ treatment (Figure 6C, middle panel), but the same As_2_O_3_ treatment markedly induced the cleaved PARP and apoptosis in GSCs (Figure 2A–2C). Interestingly, As_2_O_3_ treatment also reduced PML protein levels in NSTCs (Figure 6C, Supplementary Figure S4C) but did not show effect on cell growth of NSTCs. Taken together, the differential response of GSCs and NSTCs to the As_2_O_3_-induced PML reduction and the different PML distribution in these cell populations implicated that PML may have distinct functions via different downstream effectors in GSCs and NSTCs.

### c-Myc is the downstream effector of PML in the GSC maintenance

**Figure 7:**
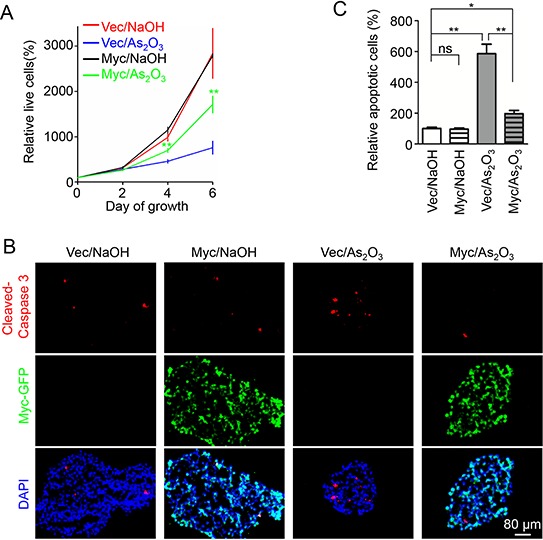
Overexpression of c-Myc partially rescued the inhibitory effects of As_2_O_3_ on GSCs **A.** Cell titer assays of GSCs overexpressing c-Myc-GFP or control vector. T387 GSCs were infected with c-Myc-GFP or control lentiviruses. 48 hours after infection, cells were planted into 96 well plates at the concentration of 2,000 cells per well. Cells were then treated with 1 μM As_2_O_3_ or 0.01N NaOH. Cell growth was monitor at the indicated time point with the cell titer Glo luminescent cell viability assay kit. A partial rescue was observed in GSCs infected with c-Myc-GFP in response to As_2_O_3_ treatment. **p < 0.01 (mean ± s.e.m.; two tailed unpaired *t*-test). **B.** Immunofluorescent staining of apoptosis in GSC spheres. T387 GSCs were cultured in 12-well plates at the concentration of 500,000 cells per well when infected with c-Myc-GFP or control lentiviruses. 48 hours after infection, GSC spheres were fixed with 4% PFA. Frozen sections of the spheres were stained with the antibody against the apoptotic marker cleaved Caspase-3 (red). Exogenous c-Myc expression was monitored by GFP signal (green). As_2_O_3_ treatment resulted in a significant increase in cleaved Caspase-3 signal in control infected GSC spheres. However, c-Myc-GFP overexpressing GSC spheres showed significant fewer cleaved Caspase-3 staining relative to the control spheres. **C.** Bar graph to show the cleaved Caspase-3 staining in control or c-Myc-GFP overexpressing xenografts treated with As_2_O_3_ or NaOH. Overexpression of c-Myc-GFP partially rescued the apoptosis induced by As_2_O_3_ treatment. *p < 0.05; **p < 0.01 (mean ± s.e.m.; two tailed unpaired *t*-test).

Differential response of GSCs and NSTCs to As_2_O_3_ treatment prompted us to explore the downstream effector of PML in GSCs. As a time course treatment of GSCs with As_2_O_3_ resulted in gradual decrease of c-Myc (Figure 2A and 2B), the downregulation of c-Myc after As_2_O_3_-induced PML degradation suggested that c-Myc may be in the downstream of PML (Figure 2A and 2B). Previous studies showed that depletion of c-Myc in GSCs caused severe cell death [40], which is consistent with the phenotypes caused by As_2_O_3_ treatment. Moreover, c-Myc is preferentially expressed in GSCs relative to NSTCs [41], whereas As_2_O_3_ treatment showed no detectable effect on NSTCs expressing low level of intrinsic c-Myc protein (Supplementary Figure S4C). These facts indicate that c-Myc is a potential effector of PML in GSCs in response to As_2_O_3_ treatment. As PML has been shown to be a major target of As_2_O_3_ in cancer stem cells in leukemia [31], we sought to determine the regulatory relationship between PML and c-Myc in GSCs. Knockdown of PML by shRNA resulted in a marked decrease of c-Myc protein levels in GSCs (Figure 6D). Furthermore, ubiquitination assay demonstrated that disruption of PML dramatically increased poly-ubiquitination of c-Myc protein and reduced c-Myc levels in GSCs (Figure 6E, Supplementary Figure S4D), suggesting that PML is required for c-Myc stabilization in GSCs. As c-Myc has been shown to interact with PML [38, 42], it's likely that the interaction between PML and c-Myc prevents ubiquitination-mediated degradation of c-Myc. Given that c-Myc is an important downstream effector of PML, the expression of c-Myc in GSCs in response to As_2_O_3_ treatment was further studied *in vivo*. Immunohistochemical staining of the GSC-derived intracranial xenografts with or without As_2_O_3_ treatment showed a marked reduction of c-Myc protein levels in the majority of tumor cells after As_2_O_3_ treatment, indicating c-Myc as a downstream effector of As_2_O_3_ treatment in GBM tumors (Figure 6F and 6G, Supplementary Figure S4E and S4F). Moreover, transient overexpression of c-Myc-GFP partially rescued the growth inhibition caused by As_2_O_3_ treatment in GSCs (Figure 7A). Immunofluorescent analysis indicated that GSCs overexpressing c-Myc-GFP had less apoptosis after As_2_O_3_ treatment (Figure 7B and 7C), highlighting the importance of c-Myc in mediating GSC resistance to As_2_O_3_ treatment. Collectively, these data demonstrated that As_2_O_3_ treatment destabilizes PML and c-Myc, which in turn impairs GSC maintenance, supporting that PML and its effector c-Myc are critical for the maintenance of the stem cell-like phenotype and tumorigenic potential of GSCs. Further investigations are required to unveil the detailed mechanisms underlying the regulation of PML on c-Myc stability.

## DISCUSSION

The high frequency of tumor recurrence in GBM patients highlights the emergent requirement for drugs targeting glioma stem cells that are believed to be responsible for therapeutic resistance and tumor propagation. Our study suggests As_2_O_3_ as a potent drug preferentially targeting GSCs in GBM tumors. As a FDA approved drug for leukemia treatment, As_2_O_3_ has a long history in clinical practices with minor and contemporary side effects [24, 43]. Furthermore, compared with temozolomide, bevacizumab and other expensive drugs, As_2_O_3_ is an affordable drug with minimal economic concerns and can benefit the maximum number of GBM patients. A study in mice has demonstrated that As_2_O_3_ is able to enter brain [44]. Although the infusion and efficiency of As_2_O_3_ in human brain tumors remained to be further determined, it's likely that As_2_O_3_ as a small molecular drug can pass through the blood brain barrier to inhibit GBM tumor growth by targeting GSCs in human brains. As_2_O_3_ may have a concentration gradient *in vivo* and tumor cells will be killed only with the effective concentrations. However, As_2_O_3_ did inhibit orthotopic tumor growth in nude mice and thus showed its potential in treating GBMs. In addition, the increased vascular permeability in GBMs relative to normal brain tissues may benefit the As_2_O_3_ infusion in the tumor region. Thus, a suitable As_2_O_3_ dosage could exert the maximal inhibitory effect on tumor with minimal side effects on normal brain tissues.

We have demonstrated that As_2_O_3_ treatment potently induced apoptosis of GSCs but showed little effect on matched NSTCs. The differential sensitivity of GSCs and NSTCs in response to As_2_O_3_ treatment may be ascribed to the destabilization of the GSC transcriptional factor c-Myc induced by As_2_O_3_-triggered PML degradation. c-Myc is a pivotal regulator controlling multiple cellular activities [45]. c-Myc is highly expressed in GSCs relative to NSTCs or normal neural progenitor cells, which provides the molecular basis for the discrimination between GSCs and NSTCs [40, 41]. However, the molecular mechanisms underlying the stabilization of c-Myc by PML in GSCs remain unclear. Previous reports described the partially localization of c-Myc in nuclear bodies along with the physical interaction between c-Myc and PML [38, 42]. It is possible that the nuclear bodies may sequester c-Myc and prevent c-Myc from the ubiquitination-mediated degradation, thereby maintain high c-Myc protein level in GSCs. On the other hand, PML nuclear bodies are believed to be transcriptional hot spots. It has been shown that PML affects transcription of several c-Myc target genes [42]. From this aspect, degradation of PML by As_2_O_3_ may affect the c-Myc regulated transcriptome in GSCs which in turn impairs the maintenance of GSCs. Although c-Myc is barely detected in neural progenitor cells, PML is found to regulate the fate of neural progenitor cells in mouse developing neocortex through affecting the subcellular distribution of the retinoblastoma protein and the protein phosphatase 1α [46]. The role of PML in human neural progenitor cells remains unknown. *In vitro* treatment of human neural progenitor cell lines with As_2_O_3_ reduced PML protein levels without obvious effects on cell growth (Supplementary Figure S5). Thus, PML in normal and tumor stem cells has distinct functions, which are likely to be mediated by different PML downstream pathways.

In previous reports using *in vitro* cultured GBM tumour-spheres, the inhibition of Notch downstream factors was observed after As_2_O_3_ treatment [32]. However, there's no evidence showing that Notch pathway could directly mediate the effects of As_2_O_3_ stimulation. On the contrary, PML is widely recognized as the major and immediate target of As_2_O_3_ [26, 28]. In fact, PML is involved in regulation of several key factors in stem cells. PML/mTOR axis plays a critical role in the maintenance of hematopoietic stem cells [31]. Interestingly, PML is also reported to contribute to GBM resistance to mTOR targeted therapies [47], suggesting its involvement in GBM tumor progression. In addition, PML expression is required for survival of hematopoietic stem cells and breast cancer stem cells through activation of PPAR-δ pathway [48, 49]. Moreover, multiple key transcriptional factors important for stem cell maintenance, including Oct4, Nanog, Stat3 and REST, have been shown to be associated with PML in stem cells [50–52]. Even the Notch pathway may be regulated by PML [53]. Thus, PML may support GSCs through multiple pathways, and degradation of PML induced by As_2_O_3_ treatment has complex destructive effects on GSC maintenance.

PML has seven known major isoforms, each containing some transcriptional variants and the corresponding protein products [36, 54]. Our results suggested expression of multiple PML isoforms in GSCs (Figure 2A and 2B). However, introduction of shRNA resistant PMLI and PMLIV, either respectively or in combination, could not rescue the GSCs depleted of endogenous PML proteins (data not shown). Since multiple, if not all, PML isoforms participate in nuclear body formation [37, 39], plural PML isoforms may function as a complex in GSCs and each of these isoforms is indispensable for GSC survival. In the meantime, PML has several kinds of post-translational modifications including sumoylation, phosphorylation, ubiquitination, and acetylation [27–29, 55]. It would be interesting to determine whether these modifications are relevant to targeting of GSCs by As_2_O_3_.

In summary, we found that As_2_O_3_ treatment can efficiently target GSCs *in vitro* and *in vivo*. Such effects can be mainly ascribed to the As_2_O_3_-induced PML reduction and subsequent c-Myc degradation mediated by poly-ubiquitination. Our findings suggest a clinical application of the well-known anti-leukemia drug As_2_O_3_ in treating the malignant brain tumors by targeting glioma stem cells.

## MATERIALS AND METHODS

### Isolation and culture of glioma stem cells (GSCs)

GSCs were isolated from primary GBM tumors or subcutaneous xenografts as previously described [3, 14, 41, 56, 57]. De-identified GBM surgical specimens were collected from Cleveland Clinic Brain Tumor and Neuro-Oncology Center in accordance with an Institutional Review Board-approved protocol. GBM tumor cells were dissociated with the Papain Dissociation System (Worthington Biochemical) and GSCs were sorted by fluorescence-activated cell sorting (FACS) using two GSC surface markers (CD15/CD133). GSCs were validated for their capacity of serial sphere formation, *in vitro* induction of differentiation and *in vivo* tumor propagation as previously described [3, 14, 57]. NSTCs were sorted CD133^−^/CD15^−^ cells from the same tumor.

### Establishment of GSC-derived orthotopic GBM xenografts and As_2_O_3_ treatment

Orthotopic GBM xenografts were derived from intracranial transplantation of GSCs as described [3, 14, 57]. Athymic female immunocompromised C57/BL6 mice of 4–6 weeks (Charles River Laboratories) were used for animal experiments. GSCs transduced with luciferase and/or shPML (P66 or P97) or shNT were injected into the right cerebral cortex at a depth of 3.5 mm. Mice were monitored by bioluminescent imaging or maintained until manifestation of neurological signs. Mouse Kaplan–Meier survival curves were made with GraphPad Prism 5 software by using Logrank two-tail analysis. All animal protocols were approved by the Animal Research Committee of the Cleveland Clinic, and all animals were housed in the Association for the Assessment and Accreditation of Laboratory Animal Care-accredited animal facility of the Cleveland Clinic.

Treatment of mice with As_2_O_3_ (Sigma-Aldrich, 202673–5G) was started on the fourth day after intracranial implantation of GSCs expressing luciferase. Mice were treated with As_2_O_3_ at a dosage of 5 μg/g (in 0.01 M NaOH) of body weight daily through intraperitoneal injection throughout the period of tumor growth. The control group mice were treated with the same volume of vehicle control (0.01 M NaOH) per day. The similar dosage of As_2_O_3_ (5 μg/g/IP/daily) applied in this treatment has been used for treating leukemia in mice by other published studies [33, 34]. No sign of toxicity was observed in As_2_O_3_ treated mice other than a slight weight loss.

### Immunoblot analysis

Immunoblotting was performed as previously described [9, 56–58]. Briefly, cells were lysed in RIPA buffer (50 mM Tris HCl pH7.4, 150 mM NaCl, 2 mM EDTA pH8.0, 1% NP-40, 0.1% SDS, 10 mM NaF, 20 mM beta-Glycerophosphate, 1 mM Na_2_VO_3_, 1 mM PMSF, protease inhibitor cocktail) and subjected to SDS-PAGE. Proteins were transferred to PVDF membrane (Biorad), blocked by 5% milk for 30 minutes, and incubated with primary antibody overnight at 4°C. Membranes were washed with TBST for 3 times and incubated with secondary antibody for 2 hours at room temperature. Membranes were then washed with TBST for 3 times and subjected to chemiluminescent substrate (Thermo Scientific). Signals were detected with ChemiDoc XRS + Imager (Biorad).

### Immunofluorescence and immunohistochemistry

Immunofluorescent staining and immunohistochemistry (IHC) were performed as described [9, 56–58]. For cultured GSCs, cells were attached to the hES-Matrix coated coverslips before staining. For immunofluorescence, Alexa-conjugated secondary (Life Technologies) antibodies were applied. For immunohistochemistry, biotinylated secondary antibodies were used together with the Vectastain ABC kit and the DAB kit (Vector Laboratories) per manufacturer's instructions.

### Antibodies and reagents

PML antibodies were purchased from Santa Cruz (sc-966) for immunofluorescent staining, or from Bethyl (A301–167A) for immunoblotting. c-Myc antibody (sc-40) and Sox2 antibody (sc-17320) were purchased from Santa Cruz. PARP antibody (9542) and cleaved Caspase-3 antibody (9661S) were from Cell Signaling. Glut1 antibody was from Thermo Scientific (PA1–37782). Arsenic trioxide was purchased from Sigma (202673–5G) and the stock solution was prepared at 100 mM as instructed by manufacturer.

### Production of shRNA Lentiviruses and knockdown

PML shRNA (shPML) and the no-targeting shRNA (shNT) control lentiviral constructs were purchased from Sigma (TRCN0000003866 for shPML-P66, TRCN0000355997 for shPML-P97, SHC002 for shNT). For virus package, 293FT cells were transfected with lentiviral construct along with psPAX2 and VSVG. 48–72 hours after transfection, viral supernatants were collected and transduced into cells. Infected cells were selected with 1 μg/mL puromycin for 2 days to get rid of uninfected cells.

### Ubiquitination assay

Ubiquitination assay was performed as previously described [58]. GSCs overexpressing shNT or shPML shRNAs were treated with the proteasome inhibitor MG132 (20 μM, Sigma-Aldrich, C2211–5MG) for 6 hours and then lysed in TritonX-100 lysis buffer, immunoprecipitated with anti-Myc agarose affinity gel (Sigma-Aldrich, A7470–1ML) and immunoblotted with an anti-ubiquitin (Biolegend, 646301) or anti-c-Myc antibody (Santa Cruz, sc-40). Briefly, cell lysates (300 μg of total protein) were incubated with 15 μL anti-Myc conjugated agarose gel with constant rotation overnight at 4°C. Immunocomplexes were washed three times with ice-cold 0.3% Triton X-100 in PBS buffer and eluted in loading buffer by boiling for 10 min, and then analyzed by immunoblotting. Proteins were resolved on NuPAGE Novex 4–12% Bis-Tris gels (Invitrogen, NP0322BOX), blotted onto polyvinylidene membranes and probed by antibodies specific to ubiquitin and c-Myc.

### Statistical analysis

The level of significance was determined by a two-tailed un-paired Student's *t*-test (bar graphs) or analysis of variance with α = 0.05 (survival curves), and then analyzed with GraphPad Prism 5 software. All quantitative data presented are the mean ± s.e.m. from at least three samples or experiments per data point.

## SUPPLEMENTARY FIGURES


