# Supplementary material for: Arsenic trioxide disrupts glioma stem cells via promoting PML degradation to inhibit tumor growth
Source: Oncotarget. 2015 Oct 14;6(35):37300–15. doi: 10.18632/oncotarget.5836 (PMC4741931; doi:10.18632/oncotarget.5836)
Supplement: Supplementary file 1 [file oncotarget-06-37300-s001.pdf]

SUPPLEMENTARY FIGURES

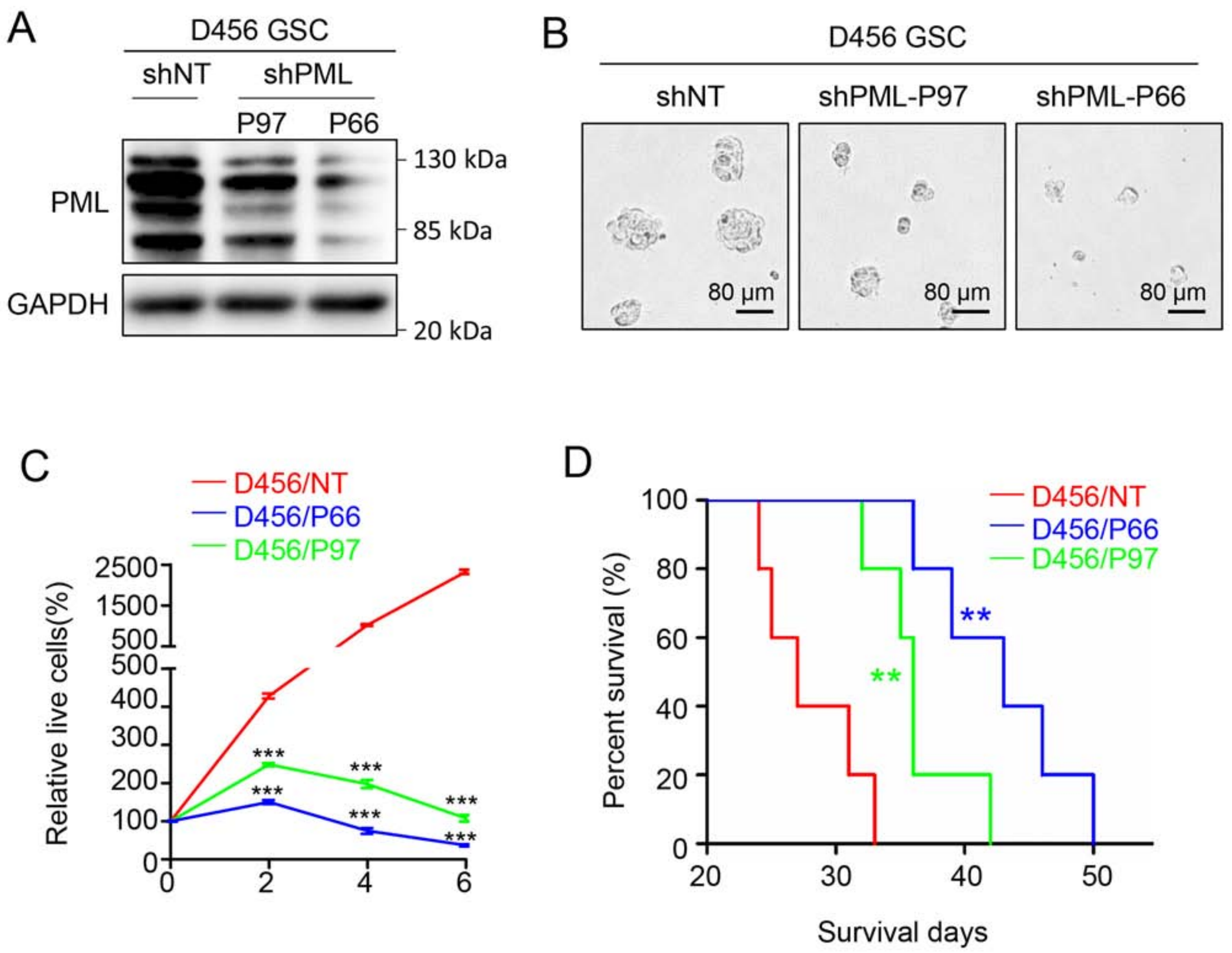

Supplementary Figure S1: Knock-down of PML in GSCs mimics As_2O_3 treatment to inhibit GSC growth *in vitro* and tumor formation *in vivo*. A. Immunoblot analysis showing the efficiency of PML knockdown in D456 GSCs by shPML lentiviruses. Both shPML-P66 and shPML-P97 infection reduced more than 80 percent of endogenous PML in GSCs. **B.** Knock-down of PML reduced GSC sphere formation. GSCs (D456) were infected with shPML or shNT lentiviruses for 24 hours, and then planted into 96 well plates at the concentration of 2,000 cells per well. Representative images showing GSC spheres 96 hours after lentiviral infection. A dramatic reduction of GSC sphere size was observed after PML knockdown. **C.** Cell titer assays showing cell growth of GSCs transduced shPML or shNT control. GSCs were infected with shPML or shNT lentiviruses for 24 hours, and then split into 96 well plates at the concentration of 2,000 cells per well. Cell titer was determined by the Glo luminescent cell viability assay kit (Promega) at the indicated time points. Disruption of PML significantly inhibited GSC growth and induced cell death. ***$p < 0.001$ (mean ± s.e.m.; two tailed unpaired t-test). **D.** Kaplan–Meier survival curves of mice bearing GBM xenografts derived from shPML- or shNT- expressing D456 GSCs. Mice intracranially implanted with shPML- and shNT-expressing GSCs were monitored and maintained until the manifestation of neurological signs. Logrank analysis revealed a significant extension of survival in groups of mice bearing shPML-GSC-derived xenografts relative to the control mice bearing shNT-GSC-derived xenografts. (n = 5 mice for each group; **: $p < 0.01$).

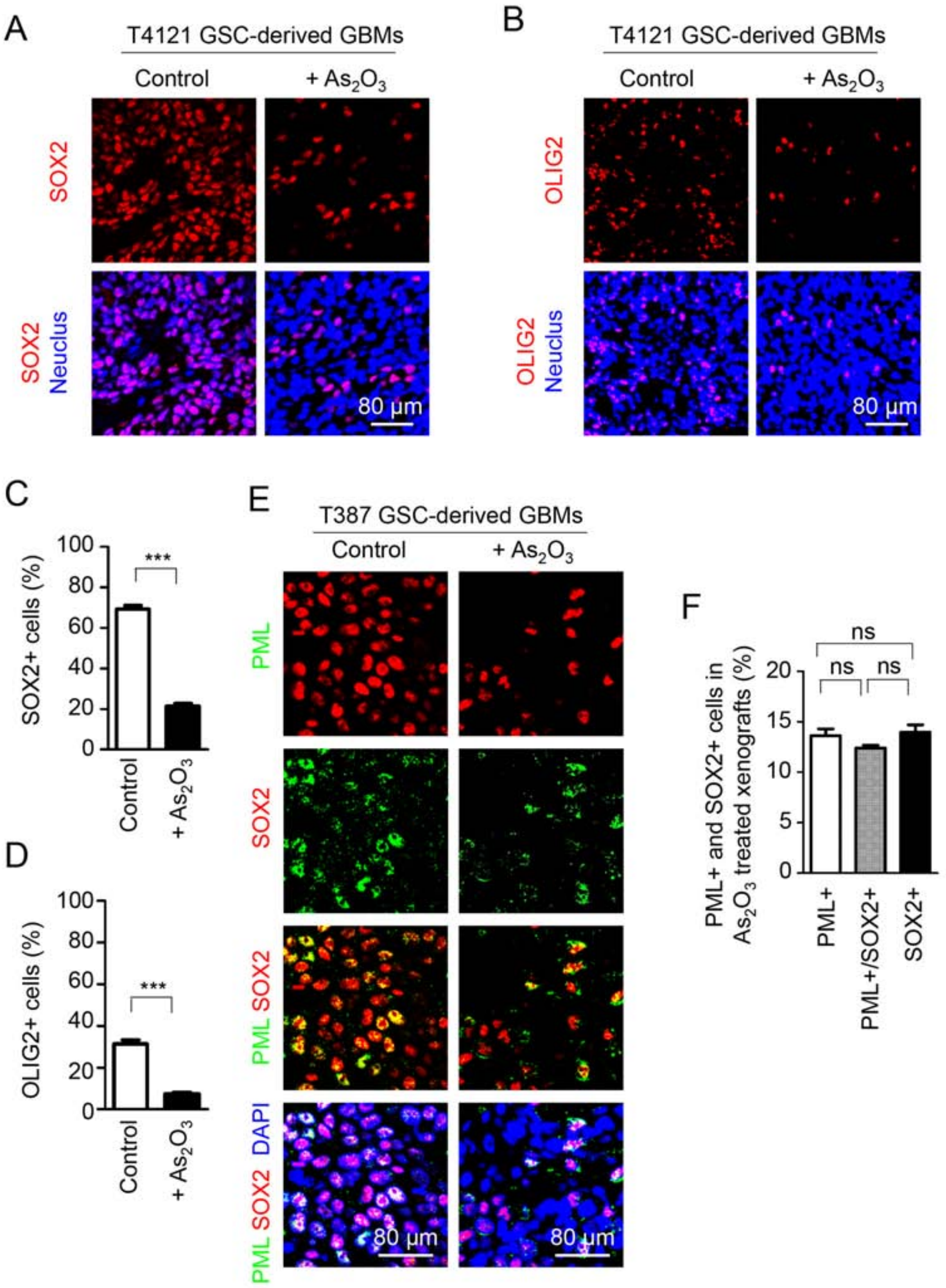

Supplementary Figure S2: As_2O_3 treatment reduced PML protein and GSC population *in vivo*. A, B. Immunofluorescent staining of the GSC markers SOX2 (A, in red) or OLIG2 (B, in red) on frozen sections of T4121 GSC-derived intracranial xenografts from mice treated with As_2O_3 or vehicle control. 4 days after GSC transplantation, mice bearing the GSC-derived xenografts were treated with As_2O_3 (5 μg/g) or vehicle control daily through IP injection for 15 days, and then mouse brains bearing the tumors were harvested and sectioned for the immunofluorescence. As_2O_3 treatment markedly reduced SOX2+ or OLIG2+ (GSCs) in the GSC-derived xenografts. **C, D**. Statistical bar graphs showing a significant decrease in SOX2+ (C) or OLIG2+ (D) population in the As_2O_3-treated xenografts relative to the control xenografts. ***$p < 0.001$ (mean ± s.e.m.; two tailed unpaired *t*-test). **E.** Immunofluorescent staining of PML (in green) and the GSC marker SOX2 (in red) in T387 GSC-derived xenografts from mice treated with As_2O_3 or vehicle control. Frozen tumor sections were counterstained with DAPI (blue). As_2O_3-treated xenografts displayed much fewer PML+ cells and SOX2+ cells (GSCs). Noticeably, most PML staining signal overlapped with SOX2 signal in GSCs in As_2O_3-treated xenografts. **F.** Bar graphs showing percentages of PML positive cells and the SOX2 positive GSC population in xenografts treated with As_2O_3. The majority of SOX2 positive GSCs remaining in the As_2O_3-treated xenografts were also PML positive.

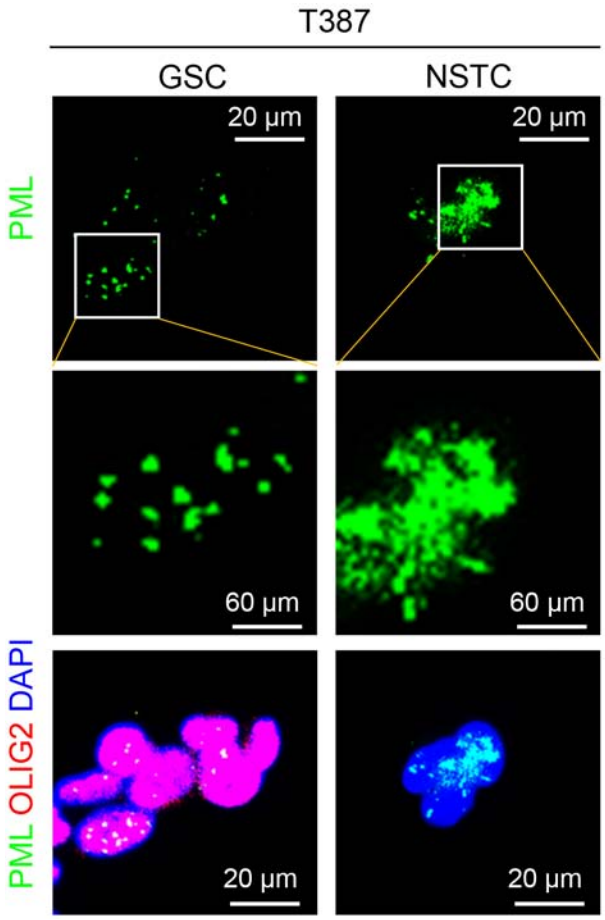

Supplementary Figure S3: GSCs and NSTCs display distinct PML distributions. Immunofluorescent staining of PML and the GSC marker OLIG2 to show pattern of PML nuclear bodies in GSCs and NSTCs. Matched GSCs and NSTCs were attached to the hES-matrix gel before staining. PML (green) appeared in punctate distributions in GSCs, while PML in NSTCs displayed a smeared distribution and formed bigger nuclear dots.

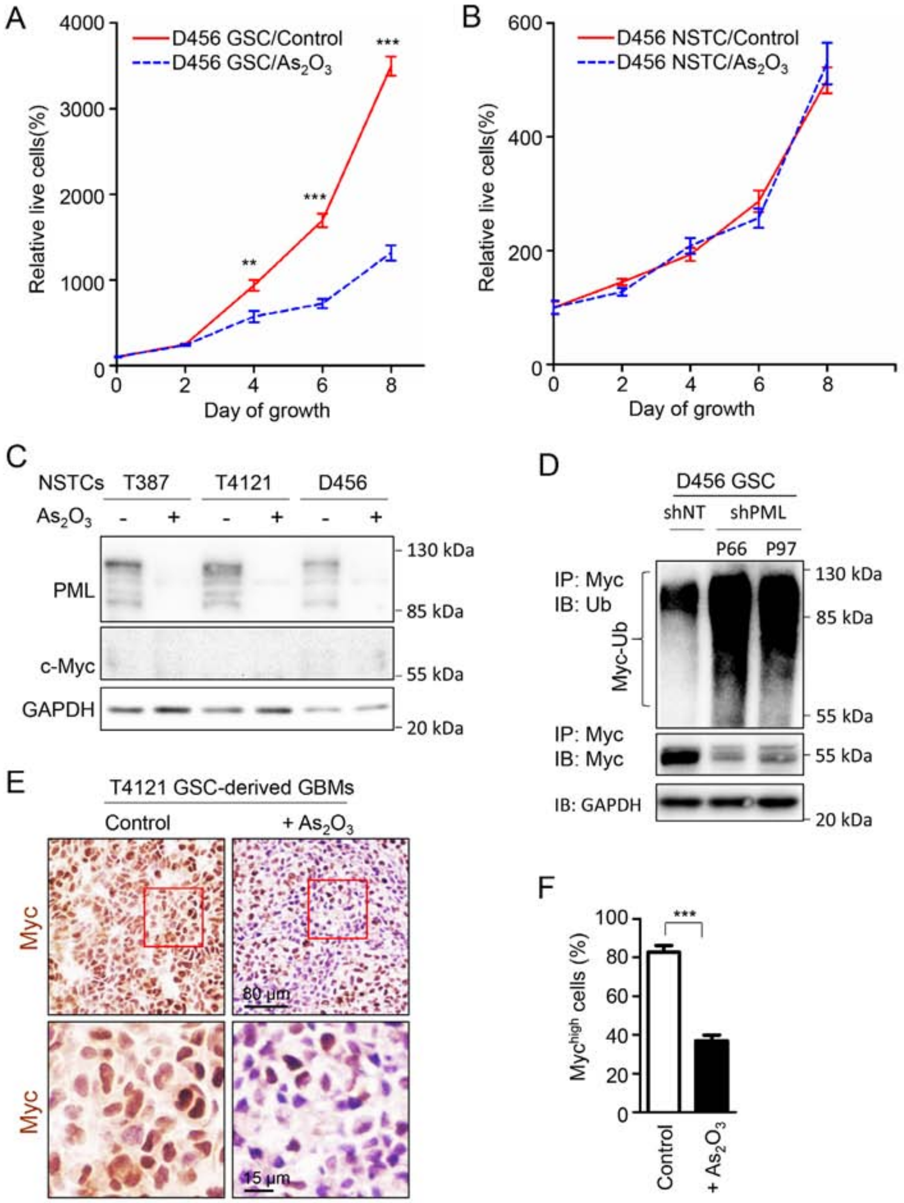

Supplementary Figure S4: c-Myc is the downstream effector of PML in GSCs that associates with the GSC-preferential effects of As_2O_3. A, B. Cell growth curves of D456 GSCs A. and matched NSTCs B. in response to As_2O_3 treatment. 2,000 GSCs or NSTCs were planted in each well of 96 well plates and then treated with 1 μM As_2O_3 or vehicle control (0.01N NaOH). Cell titer was determined by the Glo luminescent cell viability assay kit (Promega) at the indicated time points. As_2O_3 treatment significantly inhibited cell growth of GSCs but not matched NSTCs. ***p* < 0.01; ****p* < 0.001 (mean ± s.e.m.; two tailed unpaired *t*-test). **C.** Immunoblot analysis of NSTCs treated with As_2O_3. NSTCs (T387, T4121, and D456) were treated with 1 μM As_2O_3 or vehicle control (0.01N NaOH) for 48 hours. A weak intrinsic c-Myc was detected in NSTCs. As_2O_3 treatment resulted in reduced PML protein but showed no detectable effect on c-Myc protein. **D.** Ubiquitination assay of c-Myc in GSCs transduced with shPML (P66 or P97) or shNT control. GSCs (D456) were transfected with shPML or shNT through lentiviral infection for 36 hours, and then treated with 20 μM MG132 for 6 hours. Cell lysate was subjected to immunoprecipitation with the anti-c-Myc agarose beads, and the c-Myc poly-ubiquitination status was determined with anti-ubiquitin antibody. A significant increase in c-Myc polyubiquitination and a decrease of c-Myc protein levels were detected in cells transduced with shPML relative to the shNT control. GAPDH was used as the internal control for the input of immunoprecipitation (lower panel). **E.** Immunohistochemistry of c-Myc to determine the population of cells with high c-Myc expression in T4121 GSC-derived xenografts after As_2O_3 treatment. Sections of GSC-derived xenografts were stained with the antibody against c-Myc. Xenografts treated with As_2O_3 showed much weaker c-Myc staining (in brown) than those treated with the vehicle control. **F.** Bar graphs showing the population of cells with high c-Myc expression in the xenografts treated with As_2O_3 or vehicle control. A significant reduction of cells with high c-Myc expression was detected in As_2O_3-treated xenografts relative to the control xenografts. ****p* < 0.001 (mean ± s.e.m.; two tailed unpaired *t*-test).

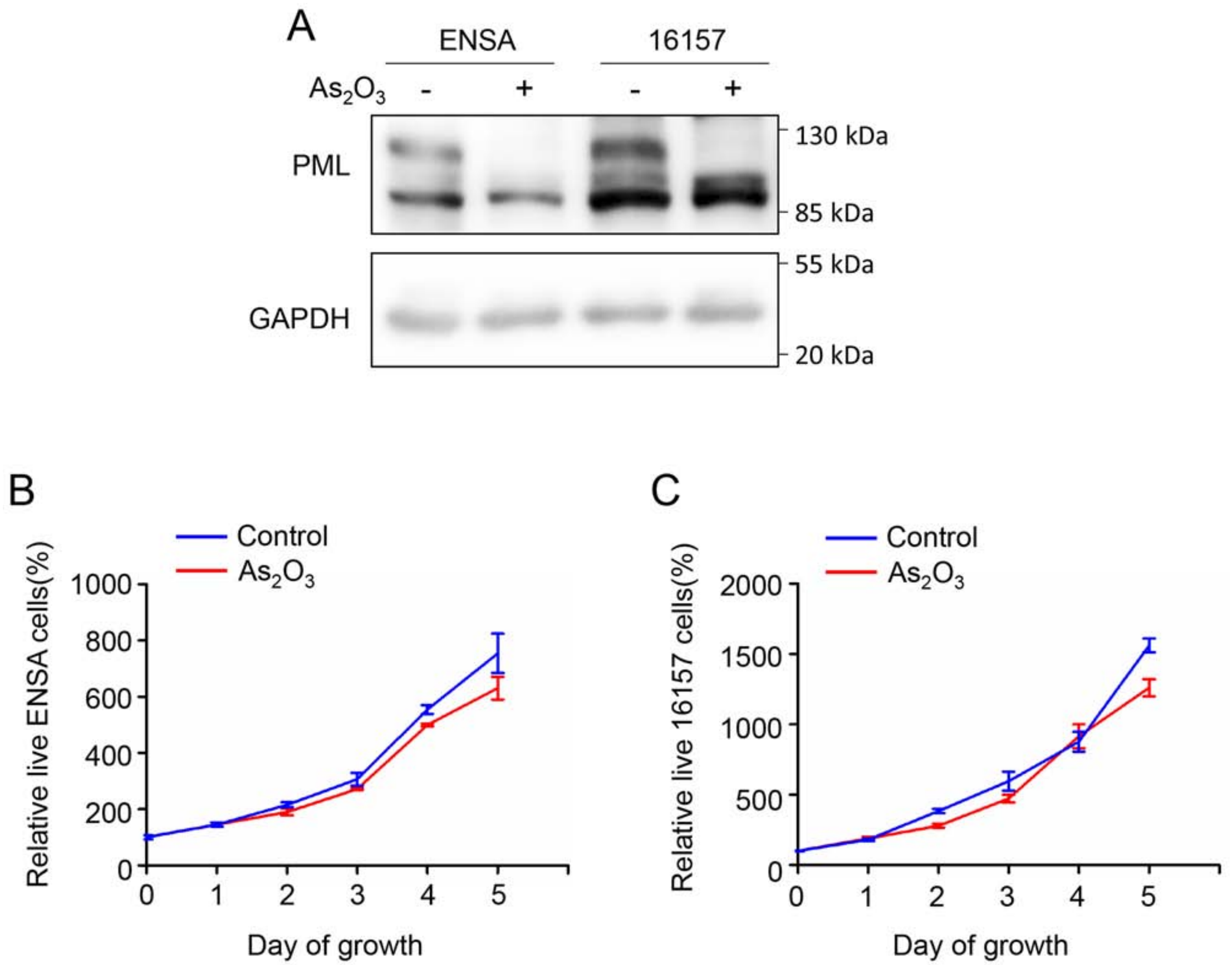

Supplementary Figure S5: As2O3-induced PML degradation has little effect on growth of human neural progenitor cells. A. Immunoblot analysis of neural progenitor cells (ENSA and 16157) treated with As_2O_3. Neural progenitor cells were treated with 2 µM As_2O_3 or vehicle control (0.01N NaOH) for 48 hours. As_2O_3 treatment resulted in reduced PML protein. **B, C.** Cell growth curves of ENSA (B) and 16157 (C) neural progenitor cells in response to As_2O_3 treatment. 1,000 cells were planted in each well of 96 well plates and then treated with 2 µM As_2O_3 or vehicle control (0.01N NaOH). Cell titer was determined by the Glo luminescent cell viability assay kit (Promega) at the indicated time points. As_2O_3 treatment showed no obvious effect on growth of neural progenitor cells.
